# Transcriptome Analysis in a Primary Human Muscle Cell Differentiation Model for Myotonic Dystrophy Type 1

**DOI:** 10.3390/ijms22168607

**Published:** 2021-08-10

**Authors:** Vanessa Todorow, Stefan Hintze, Alastair R. W. Kerr, Andreas Hehr, Benedikt Schoser, Peter Meinke

**Affiliations:** 1 Department of Neurology, Friedrich-Baur-Institute, LMU Klinikum, Ludwig-Maximilians-University Munich, 80336 Munich, Germany; vanessa.todorow@med.uni-muenchen.de (V.T.); stefan.hintze@med.uni-muenchen.de (S.H.); benedikt.schoser@med.uni-muenchen.de (B.S.); 2 Cancer Biomarker Centre, CRUK Manchester Institute, University of Manchester, Manchester SK10 4TG, UK; alastair.kerr@cruk.manchester.ac.uk; 3 Centre for Human Genetics, 93047 Regensburg, Germany; andreas.hehr@humangenetik-regensburg.de

**Keywords:** myotonic dystrophy type 1, human primary muscle cell culture, transcriptomics, splicing

## Abstract

Myotonic dystrophy type 1 (DM1) is caused by CTG-repeat expansions leading to a complex pathology with a multisystemic phenotype that primarily affects the muscles and brain. Despite a multitude of information, especially on the alternative splicing of several genes involved in the pathology, information about additional factors contributing to the disease development is still lacking. We performed RNAseq and gene expression analyses on proliferating primary human myoblasts and differentiated myotubes. GO-term analysis indicates that in myoblasts and myotubes, different molecular pathologies are involved in the development of the muscular phenotype. Gene set enrichment for splicing reveals the likelihood of whole, differentiation stage specific, splicing complexes that are misregulated in DM1. These data add complexity to the alternative splicing phenotype and we predict that it will be of high importance for therapeutic interventions to target not only mature muscle, but also satellite cells.

## 1. Introduction

Myotonic dystrophy type 1 (DM1) is a multisystemic disorder with wide ranging effects starting from skeletal muscle weakness, wasting and myotonia, cardiac arrhythmia, cataracts and insulin resistance, up to central nervous system dysfunctionality, sleep-wake cycle disturbances, endocrine dysfunction, frontal balding and a shortened lifespan [1,2,3,4]. Estimations of the prevalence of DM1 are about 1 in 8000 or fewer [5,6]. The predominant muscle involvement makes DM1 one of the most frequent muscular dystrophies in adulthood.

The occurrence, combination and severity of symptoms is highly variable and positively correlated to a pathological CTG-repeat expansion in the 3′ UTR of the *DMPK* (DM1 protein kinase) gene [7,8]. Up to 35 CTG triplets in blood derived DNA are considered normal, a repeat length between 35 and 49 is considered to be a premutation. Between 50 and ~150 repeats a mild expression of the phenotype has been observed and ~100 to ~1000 CTG repeats were identified in patients with classical DM. Repeats consisting of more than 1000 CTG-triplets usually result in congenital DM, the most severe expression of the disease [9]. The extended CTG-repeats are unstable and tend to expand further. This results in anticipation, an increase in the CTG-repeat length in offspring paralleled by earlier onset and severer disease symptoms compared to their parents [10,11,12]. The sexual inheritance also affects the severity of the disease: maternal inheritance results in more severe clinical features than paternal inheritance [13,14]. Furthermore, somatic mosaicism has been observed in DM1: a tissue-specific and age-dependent length heterogeneity of the *DMPK* CTG-repeat [15,16].

The main pathomechanism described for DM1 is RNA-toxicity caused by the formation of hairpin structures in the *DMPK* RNA transcript due to expanded CUG repeats [17,18]. The RNA gain-of-function paradigm is supported by the expression of CUG expansions independent of the *DMPK* locus in transgenic mice, which cause a severe DM1-like phenotype [19]. Regarding the mechanism by which these repeat expansions result in the DM1 phenotype, there are several proposed theories [20]. These include (i) alternative splicing of several mRNAs [21,22,23,24], (ii) altered transcriptional regulation [25,26,27], (iii) inhibited translation [28,29], (iv) repeat associated non-ATG (RAN) translation resulting in the presence of toxic peptides [30,31,32], (v) alternative polyadenylation of several mRNAs [33] and (vi) miRNA misregulation [27,34,35,36,37,38,39]. These mechanisms are not exclusive; however, they do contribute to the complex clinical phenotype.

The mechanism leading to a disbalanced splicing network originates directly from the formation of the hairpin structure in the *DMPK* mRNA, whose CUG expansion-driven secondary structure is recognized by diverse RNA-binding proteins, for instance muscleblind family members (MBNL1-3) [40,41]. The subsequent sequestration of MBNL is tantamount to its depletion from the nucleoplasm and with this, loss of function. At the same time, another splicing factor, CUGBP elav-like family member 1 (CELF1), is hyperphosphorylated and thus stabilized, leading to a gain in function [42,43]. Splicing dysregulation of several key transcripts has been shown to be involved in the development of tissue specific pathologies in DM1. Examples for genes affected by this missplicing include *CLCN1* (chloride voltage-gated channel 1) [44,45], *DMD* (dystrophin) [46], *RYR1* (ryanodine receptor 1) [47], *INSR* (insulin receptor), and *BIN1* (bridging integrator 1) [48]. Interestingly, many of the reported alternative splicing events represent a shift from adult to embryonic splicing patterning [49]. Healthy muscle tissue undergoes an MBNL/CELF-dependent, postnatal switch of alternative splicing patterns necessary to match the increasing demands of adult muscle. While MBNL1 levels are low in early embryonic stages and increase during development, CELF1 expression follows the exact opposite order [49,50]. Although MBNL and CELF1 imbalance accounts for a whole range of missplicing events in DM1, and thus have been a main scientific interest for a long time, the phenotypic presentation is unlikely to be caused entirely by those two factors alone. Indeed, a broad splicing network misregulation appears likely as the basis of DM1. Consistently, more splicing factors have been identified to regulate alternative splicing events in DM1, among them STAU1 and HNRNPA1 [51,52].

Apart from changes to the splicing pattern, which have been subject to numerous studies, there are also indications that gene expression alterations are playing a role in DM1. Examples are MBNL1 itself [53], but also various ion channels like calcium, sodium and potassium channels [53], as well as transcription factors such as MYOG, MYOD and SP1 [54]. MYOG and MYOD are both downregulated in DM1 and thus probably cause the aberrant and delayed differentiation process described in DM1 myoblast cell culture [55,56], possibly explaining poor muscle regeneration potential in patients. Furthermore, several signalling pathways are known to be affected through downregulated activity of kinases such as protein kinase B (AKT), MAPK and ERK, further contributing to the differentiation defect phenotype but also to metabolic dysregulation and autophagy [54]. Compared to the investigation of alternative splicing very little research has been focused on pathways affected by differential gene expression. A large study performed on muscle biopsies from DM1 patients and healthy controls proposed downregulation of cell adhesion and translation, as well as upregulation of mitochondrial metabolic pathways [57].

It will be of high interest for upcoming therapeutic approaches to know which defects are playing an important role in the pathomechanism at which stage of differentiation. Thus, we performed an analysis of gene expression alterations and pathway regulation in proliferating primary myoblasts as well as differentiated myotubes gained from DM1 patients and compared them to materials gained from non-disease controls.

## 2. Results

### 2.1. Differential Gene Expression in DM1 Myoblasts and Myotubes Compared to Non-Disease Controls

**Figure 1:**
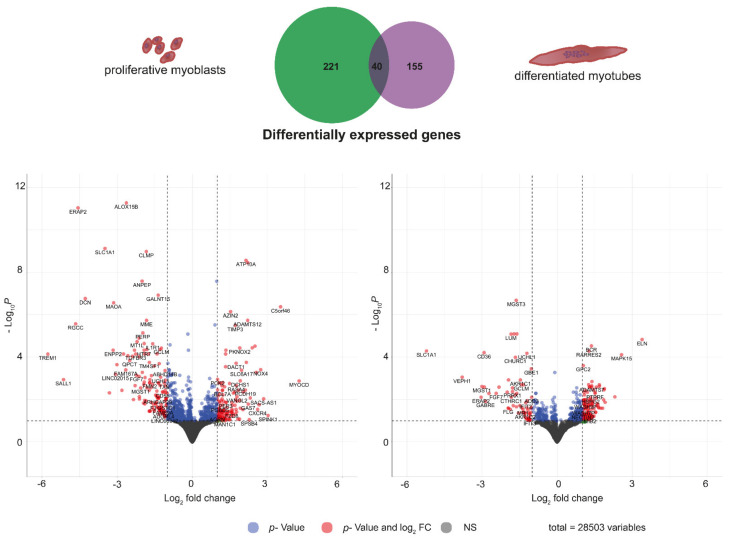
Differential gene expression between non-disease control and DM1 for proliferating myoblasts (**left**) and post-mitotic myotubes (**right**) and visualization of the number of genes with a log2 fold change > ±1 for both conditions (**at the top**).

First, DM1 samples were investigated for the typical DM1 phenotype. Cells of all DM1 tissue cultures did show nuclear MBNL1 foci (Figure S1A) and *DMPK* was slightly upregulated in myoblasts as well as myotubes (Figure S1B). To investigate differential expression of genes between non-disease control and DM1, we performed RNAseq in proliferating myoblasts and post-mitotic myotubes. To avoid contamination of the myotubes by non-differentiated cells we isolated multinucleated myotubes by selective trypsin treatment followed by sedimentation. In total, more than 28,000 genes were identified. Read counts were analysed using DESeq2. Normalized counts are listed Supplemental Table S1. We identified 261 genes in proliferating myoblasts and 195 genes in differentiated myotubes with a log2 fold change > ±1 (adjusted *p*-value < 0.1) of which 40 genes are shared between proliferating and differentiated cells (Figure 1; Tables S2 and S3), indicating differences in gene expression between non-disease controls and DM1 but also between proliferating myoblasts and post-mitotic myotubes. Gene expression changes were validated using RT-qPCR for selected genes (Figure S2). Subsequently, we performed an extensive pathway enrichment analysis using two approaches: classical gene ontology enrichment as well as gene set enrichment.

### 2.2. Pathway Enrichment Analysis: Gene Ontology “Molecular Function”

**Figure 2:**
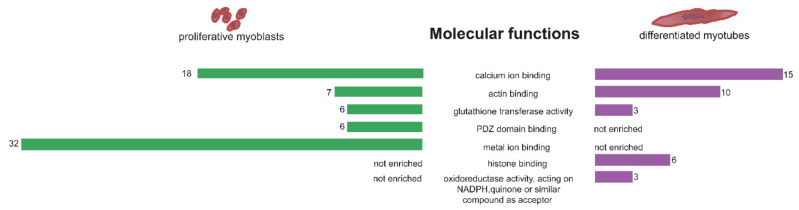
GO enrichment analysis for molecular function of genes differentially expressed between non-disease control and DM1. Significantly enriched molecular functions are shown for proliferating myoblasts (**left**, green bars) and post-mitotic myotubes (**right**, purple bars) with the number of genes in the respective molecular function displayed next to the bars.

First, we were interested in the molecular functions of the differential expressed genes. DAVID GO molecular function enrichment analysis revealed that myoblasts and myotubes share enrichment of calcium (*p*-value 0.003 and 0.01) and actin binding (0.09 and 0.002) as well as glutathione transferase activity (0.00004 and 0.04) (Figure 2). Genes found in the calcium binding cluster represent sarcomeric proteins like actinin (*ACTN4*) or calcium homeostasis factors like *ASPH*, but mostly genes related to cell adhesion and the extracellular matrix vital for successful differentiation, development of the neuromuscular junction as well as wound healing and regeneration. This includes cadherines and protocadherines (*CDH15*, *PCDH7/19*), as well as glycoproteins such as agrin (*AGRN*) and proteoglycans (*SPOCK1*, *HSPG2*). Actin binding is more enriched in myotubes than in myoblasts which can be explained by increasing demands of the cellular morphology and the development towards sarcomeric structure. Consistently, genes of the spectrin–actin complex like *SPTBN5* and *ADD3* but also heavy myosin (*MYH9*) can be found in myotubes. Glutathione transferase activity is best known for its function in detoxification and response to reactive oxygen species. Here, several subunits of the glutathione S-transferase (*GST*) are differentially expressed in both myoblasts and myotubes. Further, molecular functions only enriched in myoblasts include PDZ domain binding, indicating a role in signalling pathways, and metal ion binding including genes like Na^+^/K^+^ ATPases (*ATP1A2*, *ATP1A4*) and RAS p21 protein activator (*RASA4*). In contrast, in DM1 myotubes there is an enrichment of histone binding genes like *H4C11-15* as well as oxidoreductase activity highlighting possible changes in metabolism (Figure 2).

### 2.3. Pathway Enrichment Analysis: Gene Ontology “Signalling”

**Figure 3:**
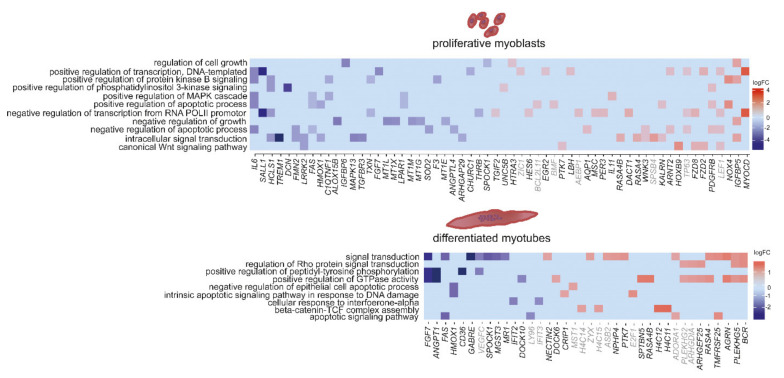
Heat maps showing differences in expression between non-disease controls and DM1 for genes linked to signalling pathways in proliferating myoblasts (**upper panel**) and differentiated myotubes (**lower panel**). Gene names displayed in grey: *p*-value < 0.1, in black: *p*-value < 0.05.

As there are reports of altered signalling in myoblasts gained from embryos with congenital DM1 [54], we were looking directly into signalling alterations. We could identify several genes involved in signalling pathways that are misregulated in DM1. This misregulation was more pronounced in myoblasts than in myotubes (Figure 3). Amongst the identified gene ontology pathways related to signalling pathways were kinase B-, MAPK-, WNT and PI3K signalling—all in myoblasts. Decorin (*DCN*) is downregulated in DM1 myoblasts (log2FC = −4.3) and is reported to positively regulate muscle differentiation and regeneration through signalling [58]. The NADPH oxidase (*NOX4*, log2FC = 2.7) generates specific reactive oxygen species (ROS) which are important signalling molecules and were shown to influence Ca^2+^ release through the ryanodine receptor (*RYR1*), among others [59]. In myotubes, identified pathways include apoptotic signalling, DNA damage response, beta-catenin TCF complex assembly as well as GTPase activity. Angiopoietin 1 (*ANGPT1*), which is downregulated in DM1 myotubes (log2FC = −2.9), plays a role in muscle regeneration, as described in murine muscle [60]. *PLEKHG5*, which is upregulated in DM1 myotubes (log2FC = 1.2), is an activator of the nuclear factor kappa B (*NFKB1*) signalling pathway, which modulates the switch between muscle proliferation and differentiation, and inhibits late-stage differentiation by silencing myofibrillar gene transcription. Misregulated genes also include the transcription factors *MYOCD* and *SALL1* (Figure 3).

### 2.4. Pathway Enrichment Analysis: Gene Ontology “Others”

**Figure 4:**
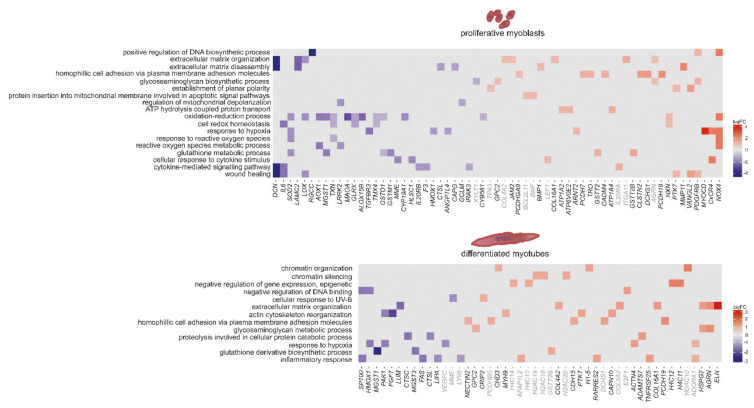
Heat maps showing differences in expression between DM1 and non-disease controls for the top enriched pathways based on GO-terms in proliferating myoblasts (**upper panel**) and differentiated myotubes (**lower panel**). Gene names displayed in grey: *p*-value < 0.1, in black: *p*-value < 0.05.

Apart from the interest in signalling, we analysed the data according to the top hits in pathway enrichment. This revealed, similar to signalling, similarities and differences between myoblasts and myotubes (Figure 4). In both, extracellular matrix organization is amongst the top hits. The above mentioned *DCN* is a member of the small leucine-rich proteoglycan (SLRP) family regulating collagen fibril assembly and thus, is a key modulator of ECM assembly. The ECM is closely connected to signalling pathways, ultimately activating myogenic factors like MYOD and MYOG, at the same time however, it is necessary for myoblast fusion. Here, genes like *DCN*, *LAMC2* (log2FC = −2.7) and *LOX* (log2FC = 1.7) which build the ECM are downregulated in myoblasts, while genes inducing ECM breakdown like *MMP11* are upregulated (log2FC = 2.4). DM1 myotubes show decreased levels of lumican (*LUM*), which belongs to the SLRP family and has similar functions to *DCN*. In both myoblasts and myotubes, collagens, cadherines and protocadherines are upregulated, while elastin (*ELN*) is strongly upregulated in myotubes only (log2FC = 3.4).

In myoblasts there are also pathways linked to mitochondria (protein insertion into mitochondrial membrane involved in apoptotic signal pathways, regulation of mitochondrial depolarization, ATP hydrolysis-coupled proton transport and the oxidation–reduction process), but also pathways linked to wound healing and cytokine-mediated signalling. In myotubes the top hits include chromatin organization and silencing, cellular response to UV-B, actin cytoskeleton organization and inflammatory response (Figure 4).

### 2.5. Splicing as the Basis of Pathway Alterations

**Figure 5:**
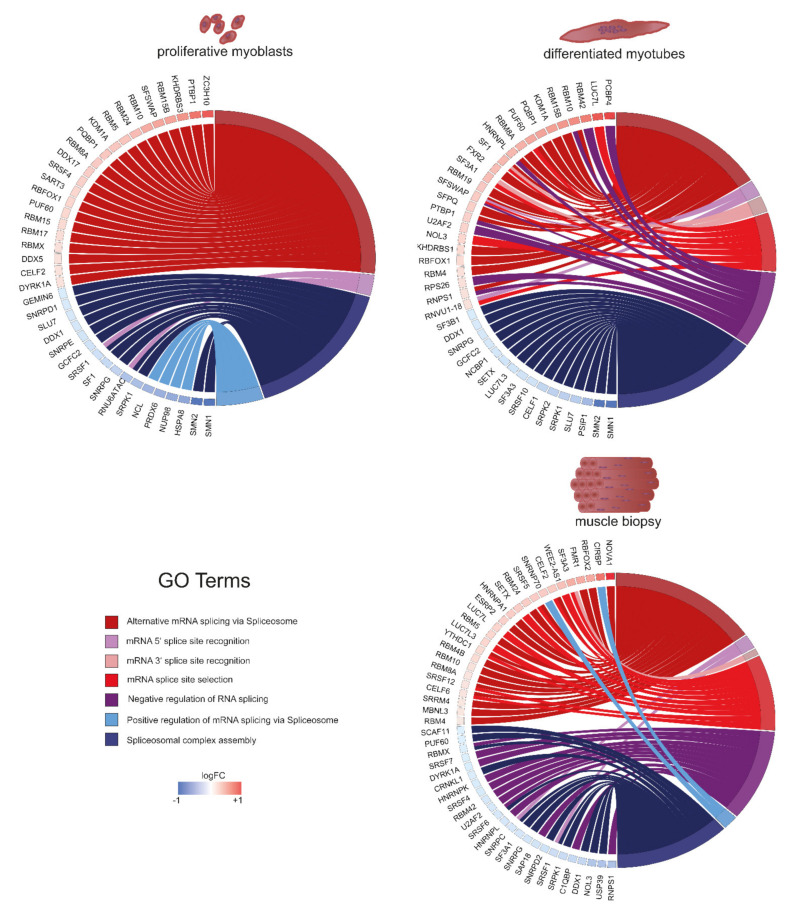
Graphs showing differences in expression between DM1 and non-disease controls for genes linked to splicing in proliferating myoblasts (**upper left**), differentiated myotubes (**upper right**), and muscle biopsies (**lower right**), sequencing data published by [57]. The colour of the boxes next to the gene name shows the level of expression change compared to controls. Genes are grouped depending on their GO-term.

Missplicing is well described for DM1. We confirmed the presence of alternative splicing events in our tissue cultures on selected genes, including *MBNL1* and *TNNT2*. *LMO7* and *TEAD1*, both encoding transcription factors, are misspliced preferentially in myotubes respectively myoblasts (Figure S3). With the specific aim of investigating splicing alterations, we performed a test for gene set enrichment (GSEA) using the fgsea R package (results in Tables S4 and S5). Splicing and especially alternative splicing is a very sensitive process at the basis of gene expression regulation which has different outcomes depending on spliceosome assembly, splice site recognition and numerous other factors (reviewed in [61]). Consistently, it was shown that expression changes of only twofold of certain splicing factors such as *SRSF1* can implicate strong effects on proliferation and apoptosis of more than twofold in cell culture [62]. We thus expect that small changes of a high number of splicing factors have broad implications, as spliceosome assembly and composition are affected. Accordingly, we analysed splicing associated genes with fold changes between 1.1 and 2/−1.1 and −2 to reveal if specific splicing pathways are affected in DM1 (Figure 5). Our data suggest that genes linked to alternative splicing via spliceosome, spliceosomal complex assembly, and mRNA 5′ splice site recognition are misregulated in both DM1 myoblasts and myotubes as well as adult muscle. Further, genes associated with positive regulation of mRNA splicing via spliceosome are downregulated in myoblasts, while in myotubes, pathways linked to mRNA splice site selection, mRNA 3′ splice site recognition, and negative regulation of RNA splicing are enriched. In muscle biopsies, we found all of these pathways affected. However, most striking was the clear cut-off between up- and downregulation. Pathways generally linked to constitutive splicing (complex assembly) are downregulated, while alternative splicing is upregulated. Notably, core components of the spliceosome machinery are underrepresented in our data and the muscle biopsy data; most splicing related genes belong to the category of accessory components important for recognition of specific splice sites rather than the splicing reaction itself. This might be a reason for the strong effects of DM1 on specific tissue types and transcripts rather than splicing in general. Members of the RNA-binding motif (RBM) family are upregulated in myoblasts, myotubes and adult muscle and play roles in alternative splicing via splice site selection. Interestingly, several studies have shown that, for example, RBM5 regulates the cell cycle and apoptosis while RBM24 is important for striated muscle differentiation, thus integrating well in our analysis so far [63,64]. Survival motor neurons 1 and 2 (*SMN1/2*) are downregulated in both proliferating and differentiated muscle cells twofold. Deficiency of *SMN1/2* is usually associated with spinal muscular atrophy; however, it was shown that low levels influence muscle differentiation and maturation through the AKT signalling pathway [65]. Similar involvements in proliferation, apoptosis and differentiation are known for many of the genes displayed in Figure 5.

## 3. Discussion

There is a plethora of data sets regarding missplicing in DM1, yet, it remains elusive which other pathomechanisms may contribute to this complex disease and how they might interconnect. We investigated gene expression in a tissue culture differentiation system using primary human myoblasts collected from DM1 patients and non-disease controls. This is highly promising in providing insight concerning the development of novel treatments and helps to answer the question of whether it will be sufficient to target mature muscle, or if it will be necessary to aim for satellite cells as well.

A general trend we observed, is that there are indeed differences in differentially expressed genes depending on the cellular differentiation status. While this is not surprising, looking specifically into affected pathways provides additional information on the disease etiopathogenesis. A possible contribution to these expression changes could be missplicing of transcriptions factors. Signalling pathways have been shown to be affected in DM1 before, explicitly in myoblasts gained from embryos with congenital DM1 [54]. Activity of the kinases protein kinase B (AKT), MAPK and ERK was downregulated in those samples. Here, we investigated myoblasts gained from adults with myotonic dystrophy and identified genes involved in the MAPK cascade, protein kinase B (AKT), WNT and PI3K signalling to be misregulated. While this supports results published on congenital DM, the lack of a misregulation of these genes in differentiated myotubes indicates the possibility of a pathomechanism specific for proliferating muscle cells.

Premature senescence has been described for DM1 primary myoblasts [66]. We previously noticed a repeat-length-dependent increase of nuclear envelope invaginations in primary human adult onset DM1 myoblasts, which was accompanied by cell cycle withdrawal of affected cells [67]. We hypothesize that these membrane invaginations are a result of missplicing events in nuclear envelope proteins like nesprin1 [68] and may cause further gene expression changes due to alterations of heterochromatin organization. Conceivably, this contributes to signalling pathways affected in proliferating myoblasts, resulting in a loss of a certain proportion of proliferating cells due to cell cycle defects. In both myoblasts and myotubes, we see strong alterations of genes involved in extracellular matrix (ECM) organization. The ECM is also closely connected to signalling pathways, ultimately activating myogenic factors like MYOD and MYOG, at the same time however, it is necessary for myoblast fusion and thus differentiation into mature myotubes.

Differentiation defects in DM1 cells [56] and patient muscle biopsies suggest a lack of fibre maturation [69]. To investigate this in more detail we decided to isolate differentiated myotubes by minimal trypsin treatment and sedimentation. While there is a potential loss of material, isolating differentiated myotubes has the benefit of avoiding contamination of undifferentiated cells. This seems especially important for a disease with known differentiation defects. The DM1 phenotype correlates approximately with CTG repeat length: the longer the repeat, the more severe the phenotype. We saw the same effect in tissue culture: for the sample with the longest repeat (DM1-3), we failed to isolate enough differentiated myotubes to get sufficient RNAseq reads for analysis. Using the data available for myotubes, we found an enrichment of genes involved in apoptosis, DNA damage and repair, histone binding, chromatin organization, and metabolic pathways. This indicates that the pathological effects in myotubes are different from myoblasts and increased stress is affecting the fitness of the cells.

Notably, we found the expression of genes linked to pathways involved in responses to reactive oxygen and inflammation being affected in proliferating and differentiated DM1 cells. This, if also affected in mature muscle fibres, might contribute to the observed fibrosis in DM1 [70].

The alternative splicing of several genes caused by MBNL/CELF misregulation is well described to drive the pathology of DM1 [71], and other splicing factors seem to be involved as well [51,52]. Importantly, our data suggest a considerably higher complexity of the splicing machinery involved than previously anticipated. Despite being rather mildly misregulated (log2FC-wise), we can see whole groups of genes linked to alternative splicing being upregulated, and the spliceosomal complex assembly downregulated in myoblasts as well as myotubes. Yet, there are differences between myoblasts and myotubes regarding genes linked to other splicing related GO-terms. In myotubes, genes involved in 5′ and 3′ splice site recognition are upregulated. A similar picture can be seen for muscle biopsies [57]. This indicates an altered formation of entire splicing complexes which are assembled specifically depending on the differentiation stage—and in turn result in a differentiation stage dependent specific misregulation in DM1. Additional work is required to further understand the mechanism of this splicing regulation.

**Figure 6:**
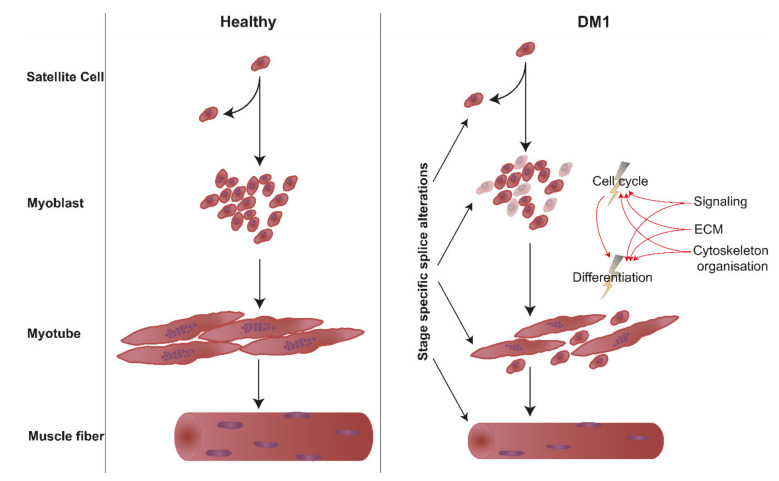
Hypothetical effect of the observed misregulated pathways on the cell cycle and differentiation leading to the DM1 phenotype.

In summary, our data suggest that developmental stage-specific missplicing of a multitude of genes affects different pathways in a stage-dependent manner, and moreover, that these pathways are highly interconnected (Figure 6). Based on our observations, we propose that missplicing-induced alterations of gene expression and signalling result in proliferation, ECM and cytoskeleton misregulation in DM1 myoblasts, ultimately leading to both cell cycle and differentiation defects, the first of which again influences the second as fewer myoblasts are present for fusion. In DM1 myotubes, which consequently are smaller and fewer in number compared to their healthy counterparts, we already observe inflammatory responses, metabolic defects and DNA damage responses, forecasting the mature DM1 phenotype. We conclude that it is vital for future therapeutic interventions to target muscle stem cells, which are the precursor cells for myoblasts and myotubes, in addition to mature muscle.

## 4. Materials and Methods

### 4.1. Culture of Primary Skeletal Muscle Cells

Primary human myoblasts were cultured in Skeletal Muscle Growth Medium (PELOBiotech, Munich, Germany) in an incubator at 37 °C and 5% CO_2_. The growth medium was supplemented with GlutaMax, 40 U/mL Penicillin, 0.04 mg/mL Streptomycin, and SkMC Supplement (PELOBiotech, Munich, Germany). Myoblasts were kept from reaching confluency to avoid differentiation. Passage numbers were matched for controls and patient cells.

For differentiation, confluent myoblasts were cultivated for 7 days in DMEM containing 5% horse serum under the same environmental conditions.

### 4.2. Patients and Non-Disease Controls

Human control and patient materials (Table 1) were obtained with written informed consent of the respective donors from the Muscle Tissue Culture Collection (MTCC) at the Friedrich Baur Institute (Department of Neurology, LMU Klinikum, Ludwig Maximilians University, Munich, Germany). Ethical approval for this study was obtained from the ethical review committee at the Ludwig Maximilians University, Munich, Germany (IRB-no. reference 45-14; 4 August 2014).

### 4.3. Myotube Separation

Myotubes were separated from undifferentiated, mononucleated cells by a minimal trypsin treatment to detach the myotubes. This was followed by gentle centrifugation to sediment myotubes and separate them from remaining mononucleated cells. All steps were controlled by light microscopy to observe the success of the separation.

### 4.4. RNA Isolation

After two washing steps with 1x PBS Trizol^®^ was added to the samples (material gained from myotube separation respectively plates with myoblasts). The Trizol^®^/sample mixture was used directly to isolate the RNA using the Dircet-zol^TM^ RNA MiniPrep Plus Kit (ZYMO Research, Freiburg, Germany).

### 4.5. RNA Sequencing

Library preparation was performed using the TruSeq Stranded mRNA Kit (Illumina, San Diego, CA, USA) with the TruSeq RNA Single Index Set A (Illumina) according to the recommended procedure. Quality and size distribution of the generated libraries has been validated using an Agilent 2100 bioanalyzer with high-sensitivity DNA chip (Agilent, Santa Clara, CA, USA) and DNA yield was measured using the Qubit dsDNA HS Assay Kit followed by pooling of the libraries in batches of 12 and seqencing of 1.2 pM pooled library on an Nextseq 500/High Output Flow Cell Cartridge using an paired end, 2 × 76 reads, single index protocol.

### 4.6. RT-qPCR

Reverse transcription of RNA was performed using the QuantiTecT Reverse Transcription Kit (Qiagen, Venlo, Netherlands). For the reaction we used the SYBR^®^ Green Master Mix (Bio-Rad, Hercules, CA, USA) and samples were run and measured on CFX Connect^TM^ (Bio-Rad). As reference gene, *Ap3d1* was used as it has been shown to be a suitable reference gene in normal and dystrophic cell culture models of myogenesis [72]. Samples were analysed using the delta Ct method and samples were normalized to the mean of the controls. Primer sequences are listed in Table S6.

### 4.7. Immunofluorescence Staining and Microscopy

Myoblasts were fixed with −20 °C cold methanol. Following primary antibodies were used for staining: MBNL1 (HPA035098, atlas antibodies), Emerin 5D10 (provided by Glenn E. Morris). Secondary antibodies were Alexa Fluor conjugated and generated in donkey with minimal species cross-reactivity. For DNA visualization DAPI (4′,6-diamidino-2-phenylindole) was used.

Images were obtained using an Olympus IX83 inverted microscope equipped with a 100× objective and a digital camera (UC90, Olympus, Shinjuku, Japan).

### 4.8. Quantification of Splicing Alterations

Splice events covering exon–exon junctions were visualized using Sashimi plots in the IGV genome browser. Reads spanning exon–exon junctions for the respective splice events were counted for controls and DM1 samples and visualized as percentage.

### 4.9. Bioinformatics

Reads were aligned to human genome assembly hg38 using STAR (version 2.6.1b) [73] and processed using deeptools (version 3.0.2) [74]. All subsequent analysis steps were conducted in R 4.0.4. BAM files were counted using featureCounts and then analysed with DESeq2 [75,76]. Normalized reads were used for quality control checks (Figures S4 and S5) which highlighted the relatively high variance of biological replicates, especially those of non-disease controls, ultimately leading to a lower number of genes with high significance and with this, a loss of biologically relevant information. Principal component analysis (PCA, Figure S4) further shows that there are more differences between undifferentiated and differentiated than between disease and non-disease samples, which is to be expected. Moreover, biological replicates more-or-less group together as expected. We decided to screen for biological meaningful results using standard approaches (DESeq2 + DAVID GO enrichment analysis) as well as an internal ranking as used in gene set enrichment analysis (GSEA, Tables S6 and S7). For both, we used the log2 fold changes generated in DESeq2 transformed with lfcShrink to account for high log2 fold changes [77]. For standard analysis, we set log2FC > ±1 and *p*-value < 0.1 to be significantly differentially expressed between samples, the latter of which can be reasoned by using biological rather than technical replicates resulting in higher variance, naturally. For splicing analysis however, we used log2FCs between −1 and 1, but higher than 0.1. Visualization was performed using packages listed in Supplemental Table S7.

### 4.10. Muscle Biopsy Data Analysis

Muscle biopsy data for DM1 and controls was previously published by [57]. Raw sequencing data for four controls and three DM1 samples was downloaded from GEO (GSE86356) and analysed by following the same workflow for myoblasts and myotubes described above, including all parameters and cut-offs. Accordingly, data quality was confirmed through dispersion estimation and PCA (Figures S6 and S7). Additionally, normalized count reads are listed in Table S8, while DESeq2 and fgsea results can be found in Tables S9 and S10.

## Figures and Tables

**Table 1:** List of patient and control tissue cultures.

	Phenotype	Age at Biopsy	Sex	CTG-Repeat Length
DM1-1	DM1	27	M	400–600
DM1-2	DM1	34	M	240–430
DM1-3	DM1	29	F	800–1500
Control-1	---	32	M	n.d.
Control-2	---	49	F	n.d.
Control-3	---	49	F	n.d.

n.d. = not determined; M = male; F = female.

## Data Availability

Processed data on DM1 and control myoblasts and myotubes can be found in the Supplemental Materials. Raw data are available on request from the corresponding author. Raw data on DM1 and control muscle biopsies are publicly available at GEO (GSE86356).

## Supplementary Materials

The following are available online at https://www.mdpi.com/article/10.3390/ijms22168607/s1.
