# Supplementary figures and images for: Transcriptome Analysis in a Primary Human Muscle Cell Differentiation Model for Myotonic Dystrophy Type 1
Source: Int J Mol Sci. 2021 Aug 10;22(16):8607. doi: 10.3390/ijms22168607 (PMC8395314; doi:10.3390/ijms22168607)

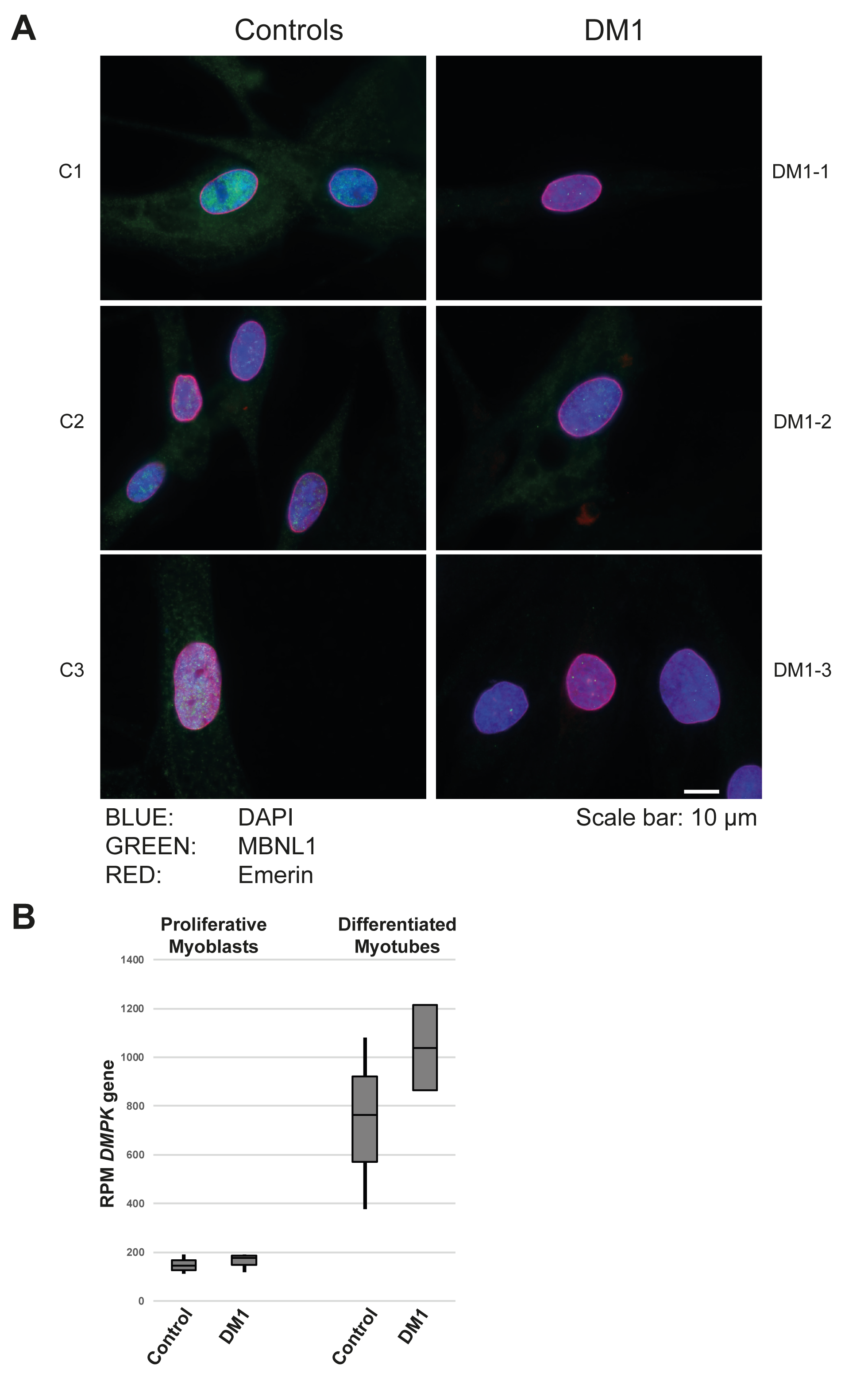

Supplement: Supplementary file 1 [file ijms-22-08607-s001.zip › Figure S1.tif]

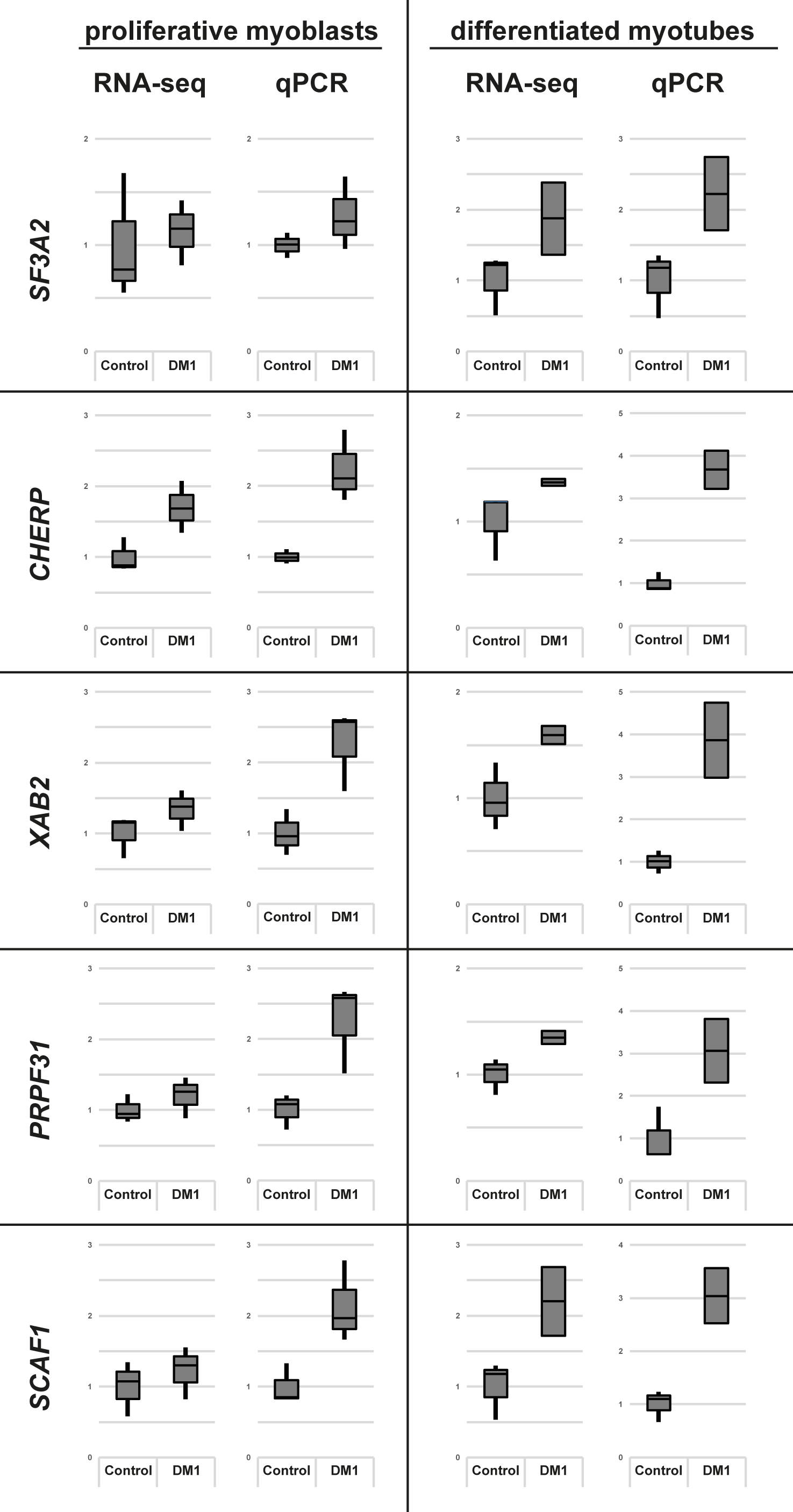

Supplement: Supplementary file 1 [file ijms-22-08607-s001.zip › Figure S2.tif]

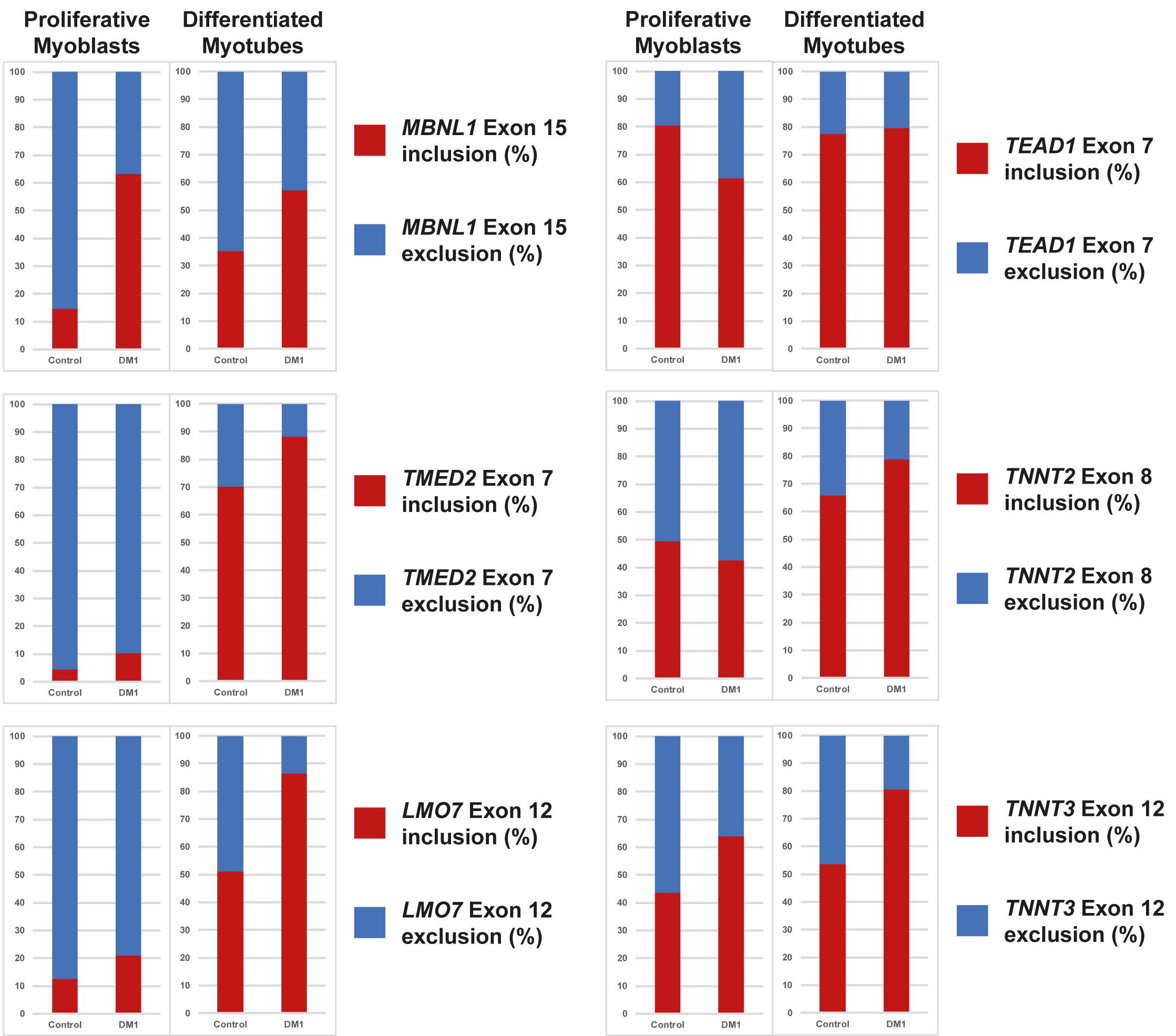

Supplement: Supplementary file 1 [file ijms-22-08607-s001.zip › Figure S3.tif]

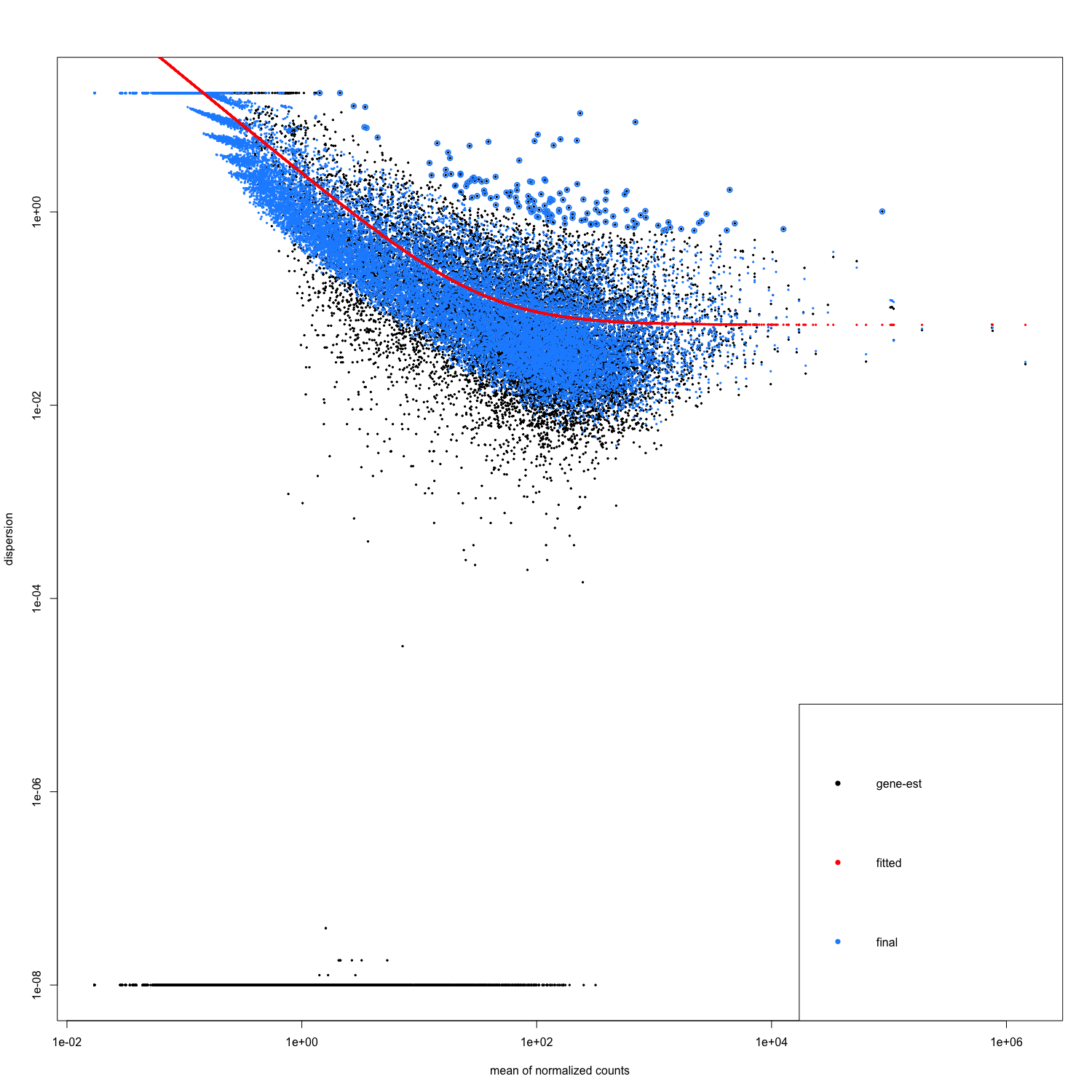

Supplement: Supplementary file 1 [file ijms-22-08607-s001.zip › Figure S4.tiff]

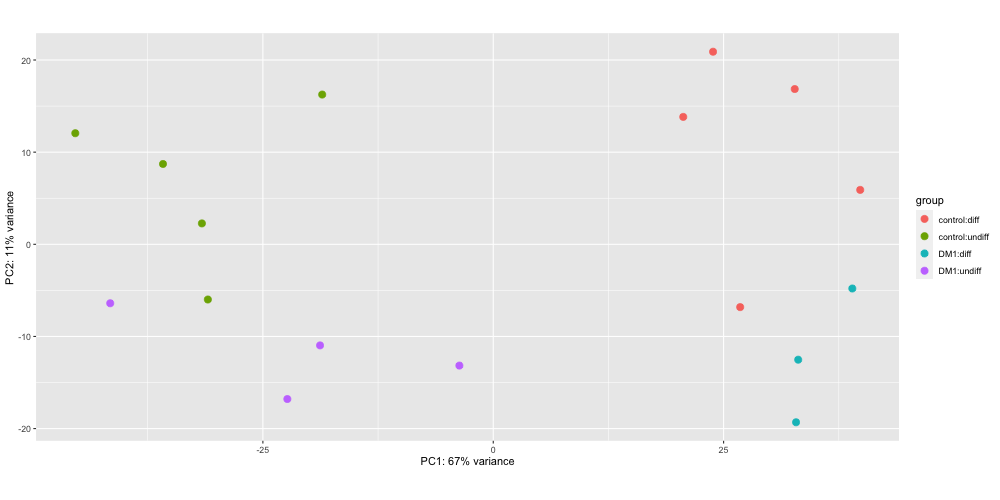

Supplement: Supplementary file 1 [file ijms-22-08607-s001.zip › Figure S5.tiff]

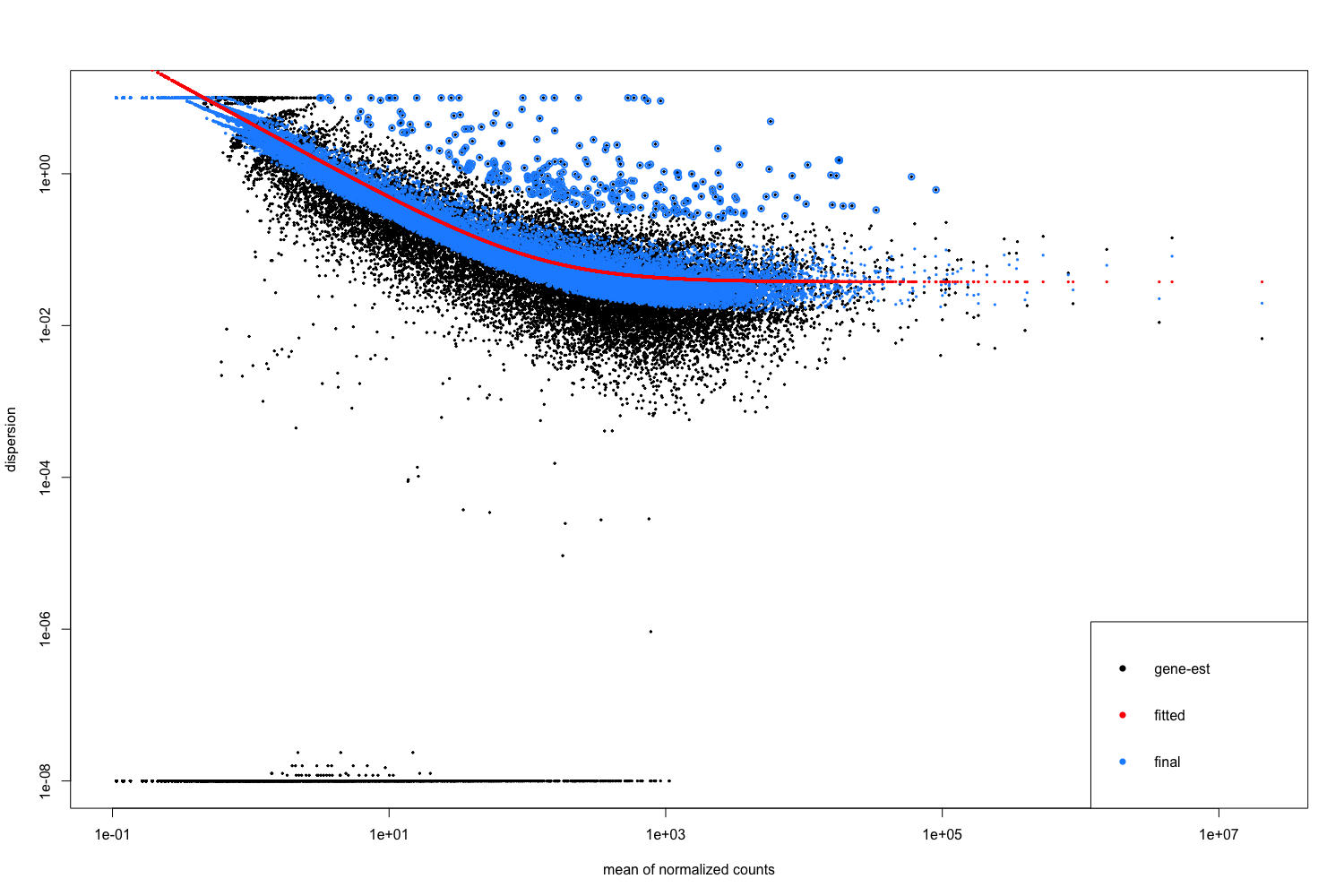

Supplement: Supplementary file 1 [file ijms-22-08607-s001.zip › Figure S6.tiff]

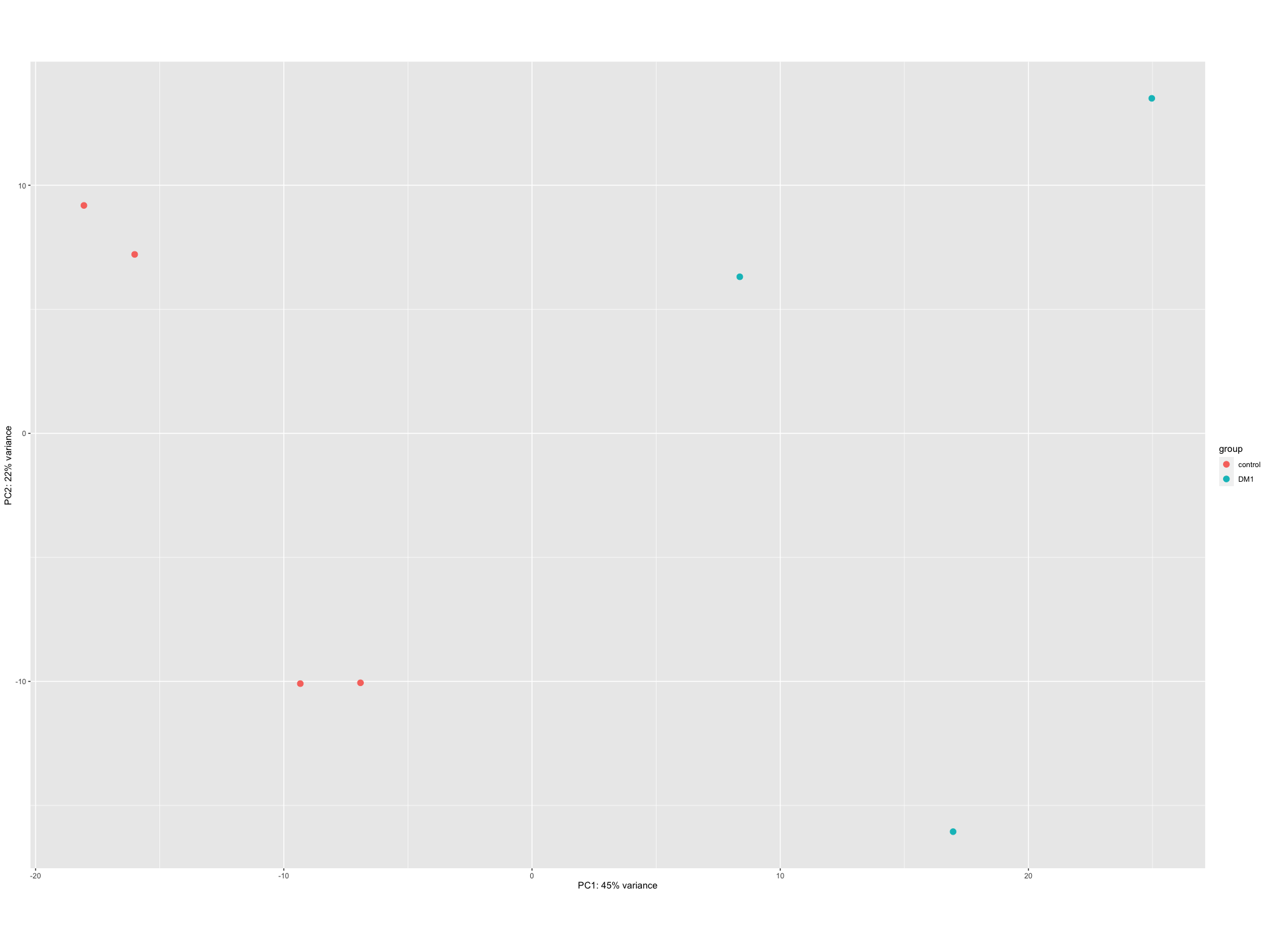

Supplement: Supplementary file 1 [file ijms-22-08607-s001.zip › Figure S7.tiff]
